# Animal based low carbohydrate diet is associated with increased risk of type 2 diabetes in Tehranian adults

**DOI:** 10.1186/s13098-020-00596-2

**Published:** 2020-10-07

**Authors:** Sohrab Sali, Hossein Farhadnejad, Golaleh Asghari, Farshad Teymoori, Parvin Mirmiran, Abolghassem Djazayeri, Fereidoun Azizi

**Affiliations:** 1 Nutrition and Endocrine Research Center, Research Institute for Endocrine Sciences, Shahid Beheshti University of Medical Sciences, P.O. Box: 19395-4741, Tehran, Iran; 2 Department of Nutrition, School of Public Health, Iran University of Medical Sciences, Tehran, Iran; 3 Department of Community Nutrition, School of Nutritional Sciences and Dietetics, Tehran University of Medical Sciences, Tehran, Iran; 4 Endocrine Research Center, Research Institute for Endocrine Sciences, Shahid Beheshti University of Medical Sciences, Tehran, Iran

**Keywords:** Low carbohydrate diet, Carbohydrate, Diabetes mellitus, Adult

## Abstract

**Background:**

To investigate the association of low carbohydrate diet (LCD) score with the risk of type 2 diabetes among adults.

**Methods:**

This cohort study was conducted on 4356 healthy participants aged ≥ 19 years old, who were followed-up for a mean duration of 3 years within the framework of the Tehran Lipid and Glucose Study. LCD score was calculated using a food frequency questionnaire according to intake of carbohydrate, protein, and fat at baseline. Diabetes was defined according to the criteria of the American Diabetes Association. Multivariable logistic regression models, adjusted for potential confounders, were used to estimate risk of diabetes across quartiles of LCD score.

**Results:**

Mean ± SD age of the study participants (44.4% men) was 40.5 ± 13.0 years. The median (25–75 interquartile range) of LCD score was 17.0 (12.0–21.0) and after a 3 year follow-up period, 123 (2.8%) incident cases of diabetes were ascertained. After adjustment for confounding variables, including age, sex, smoking status, physical activity, total calorie intake, saturated fatty acid, waist circumference, educational level, and family history of diabetes, the multivariable-adjusted ORs (95% CIs) of type 2 diabetes, comparing the highest with the lowest quartiles, were 2.16 (1.16–4.04) for total LCD score (P-value = 0.015), 1.81 (1.06–3.11) for animal-based LCD score (P-value = 0.029), and 1.47 (0.85–2.52) for plant-based LCD score (P-value = 0.160).

**Conclusion:**

Our findings suggest that a higher adherence to LCD, mostly with higher intakes of protein and fat from animal-source foods, can increase the incidence of diabetes; however, a plant-based low-carbohydrate dietary pattern is not significantly associated with risk of type 2 diabetes.

## Background

Diabetes mellitus is a serious life-threatening health problem characterised by high blood glucose levels. Accordingly, this metabolic disease has some major negative effects on quality of life and also increases healthcare costs, comorbidities, and mortality [1, 2]. Increasing evidence showed protective effects of lifestyle modifications such as diet and physical activity modification and weight reduction as applicable strategies to reduce or delay the potential risk of diabetes [3–5]. Similar to most people in the Middle East and North Africa (MENA) region, the Iranian population traditionally consume large amounts of carbohydrate foods including refined grains, rice, and potato as the main sources of energy intake in their diet [6]; therefore, investigation of the macronutrients effect in the form of a dietary pattern such as the low-carbohydrate diet (LCD) score on the risk of chronic diseases, including metabolic syndrome, diabetes, and cardiovascular diseases (CVDs) are reasonable and warranted [7–9].

The association of LCD with risk of diabetes has been assessed in some studies with controversial findings, which were mostly conducted in Western societies [10–14]. Studies on Chinese population [12], middle-aged American men [10], and women with history of GDM [13] revealed that a high score of LCD is associated with the increased risk of diabetes, particularly with high protein and fat intakes from animal-source foods. However, among Japanese women, it was observed that greater adherence to LCD was associated with the decreased risk of diabetes [14]. On the other hand, the Halton et al. study suggested that dietary pattern with lower carbohydrate and higher fat and protein are not associated with the risk of type 2 diabetes in women [11].

Since the population of MENA region, traditionally have greater adherence to LCD with higher content of simple sugars, the potential association of a dietary pattern based on the lower intakes of carbohydrate with the risk of diabetes is currently unclear in this region. Therefore, in this study, we aimed to investigate the association between greater adherence to overall LCD score, animal-based LCD score, and plant-based LCD score, and risk of type 2 diabetes among Tehranian adults.

## Methods

### Study participants

The present study was conducted within the framework of the Tehran Lipid and Glucose Study (TLGS), which conducted to determine the risk factors for non-communicable diseases among a representative urban population of Tehran, including 15,005 participants aged ≥ 3 years. The TLGS is an ongoing population-based prospective study initiated in 1999 (baseline phase) and its data are being collected prospectively at 3-year intervals; details of the TLGS have been previously reported [15].

**Fig. 1:**
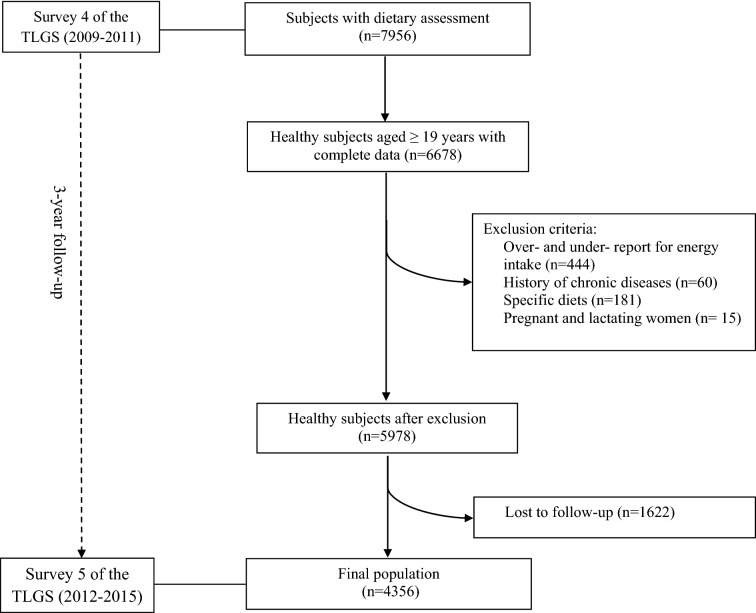
Flow chart of the Tehran Lipid and Glucose Study (TLGS) participants

In the fourth phase of the TLGS (2009–2011) conducted on 12,523 participants, 7956 subjects were randomly selected for dietary assessment. For the current study, a total of 6678 individuals, aged ≥ 19 years, with complete data in the fourth survey of the TLGS (baseline examination) were enrolled and then followed up to the fifth survey (outcome examination) with a median follow-up of 3 years. Participants with a history of myocardial infarction, stroke, and cancer (n = 60), those who reported daily energy intakes outside the range of 800–4200 kcal/day (n = 444), those on specific diets (n = 181), and pregnant and lactating women (n = 15) were excluded from this study. Finally, 5978 participants were followed up to the fifth phase of the TLGS (2012–2015), with a 3-year mean period from the baseline examination. After excluding the participants who were missed to follow up (n = 1622), the final analyses were conducted on 4356 adults after a 3-year follow-up (Fig. 1).

### Dietary assessment

Dietary intakes of the participants over the previous year were determined using a valid and reliable 147-semi-quantitative food frequency questionnaire (FFQ) [16]. Trained dieticians, during face-to-face interview asked participants to designate their consumption frequency for each food item on a daily, weekly or monthly basis during the previous year. Portion sizes of consumed foods, reported in household measures were then converted to grams. The United States Department of Agriculture (USDA) food composition table (FCT) was used to compute energy and nutrients content of food items. Local food items that were not available in USDA FCT, was analysed using the Iranian FCT.

To calculate LCD scores, as described by Halton et al., the individuals were divided into 11 strata according to each of carbohydrate, protein, and total fat as a percentage of energy intakes [11]. The percentage of energy consumed were used instead of absolute intake to reduce bias due to underreporting of food consumption and to represent dietary composition. To determine the carbohydrate score, all carbohydrate sources, including refined grains, whole grains, simple sugars, fruits, vegetables, legumes, and etc. were considered. For carbohydrate intake, participants in the highest strata received 0 points, individuals in the next strata received 1 point, and so on, down to adults in the lowest strata, who received 10 points. For protein, and fat the order of the strata was reversed; those with the highest protein and fat intakes received 10 points and those with the lowest protein and fat intakes received 0 points. The scores for each macronutrient were then summed to create LCD diet score, which ranged from 0 (the lowest fat and protein intakes and the highest carbohydrate intakes) to 30 (the highest protein and fat intakes and the lowest carbohydrate intakes). Therefore, the higher LCD diet score indicates the higher adherence of participant to the pattern of a low-carbohydrate diet.

Two additional LCD scores, including animal-based LCD score (based on the percentage of energy from carbohydrate, animal protein, and animal fat) and plant-based LCD score (according to the percentage of energy from carbohydrate, vegetable protein, and vegetable fat) were also calculated. To calculation of these scores, the individuals were divided into 11 strata according to each of carbohydrate, animal-plant- based protein, and animal-plant-based fat as a percentage of energy intakes. For animal-based LCD score, animal fat and animal protein intake was scored as 10, 9, 8, 7, 6, 5, 4, 3, 2, 1, and 0 respectively while the carbohydrate intake was scored as 0, 1, 2, 3, 4, 5, 6, 7, 8, 9, and 10 respectively in participants. For plant-based LCD score, we assigned 0–10 scores for increasing intake of plant fat, 0–10 scores for increasing intake of plant protein, and inversely, 10–0 scores for increasing intake of carbohydrates.

### Physical activity assessment

Physical activity was assessed using a Modifiable Activity Questionnaire (MAQ), which previously modified and validated for Iranian population [17]. Individuals were asked to report and identify the frequency and time spent on activities of light, moderate, hard, and very hard intensity during the past 12 months, according to a list of common activities of daily life; physical activity levels were expressed as metabolic equivalent hours per week (MET-h/wk).

### Clinical and biological assessments

A trained interviewer used a pretested questionnaire to collect data on age, sex, medical history, medication use, and smoking habits. The participant’s weight was measured and recorded in light clothing, without shoes or socks, using a digital scale with an accuracy of up to 100 g. Height was measured in a standing position without shoes, using a stadiometer to the nearest 0.1 cm. Body mass index (BMI) was computed as weight (kg) divided by height (m^2^). Waist circumference was measured at the abdominal level, at the umbilical level, over light clothing, without any pressure to body surface, using an unstretched shape tape meter. Measurements were recorded to the nearest 0.1 cm. Arterial blood pressure was measured twice on the right arm, using a mercury sphygmomanometer and the Korotkoff sound technique with an accuracy of 2 mmHg for each participant after a 15-min rest while sitting on chair with a minimum interval of 30 s; the average of the two measurements was considered to be the final pressure.

A blood sample was taken in a sitting position after 12–14 h of overnight fasting according to the standard protocol and centrifuged within 30–45 min of collection. All blood analyses were performed at the TLGS research laboratory on the day of blood collection. The samples were analysed using the Selectra 2 auto-analyzer (Vital Scientific, Spankeren, and Netherlands). Fasting plasma glucose (FPG) was measured using an enzymatic colorimetric method with glucose oxidase. Both inter- and intra-assay coefficient variations were 2.2% for FPG. For the oral glucose tolerance test, 82.5 g of glucose monohydrate solution (equivalent to 75 g anhydrous glucose) were administered orally to subjects, aged > 20 years, except for those with diabetes and taking medication. A second blood sample was taken 2 h after glucose ingestion. These analyses were performed using commercial kits (ParsAzmoon, Tehran, Iran). We measured the serum triglyceride (TG) concentration by enzymatic calorimetric method with glycerol phosphate oxidase. Inter- and intra-assay coefficients of variations (CV) for TGs were 0.6% and 1.6%, respectively. We assessed total cholesterol (TC) with cholesterol esterase and cholesterol oxidase by the enzymatic colorimetric method. High density lipoprotein cholesterol (HDL-C) was measured with phosphotungstic acid after precipitation of Apolipoprotein β. Inter- and intra-assay CVs for both TC and HDL-C were 0.5% and 2%, respectively. Low density lipoprotein cholesterol (LDL-C) was calculated from the serum and TC, TG and HDL-C concentrations are expressed in mg/dl using the Friedewald formula. These analyses were performed using commercial kits (Pars Azmoon, Tehran, Iran).

### Definitions

Based on JNC8 criteria, hypertension was defined as SBP/DBP ≥ 140/90 mmHg for individuals, aged < 60 years and as SBP/DBP ≥ 150/90 mmHg for those aged ≥ 60 years or taking antihypertensive medications for a definite diagnosis of hypertension [18]. Diabetes was defined according to the criteria of the American Diabetes Association (ADA) as FPG ≥ 126 mg/dl or 2-h post 75 g glucose load ≥ 200 mg/dl or being on oral hypoglycemic medication [19].

### Statistical analysis

All statistical analyses were performed using the Statistical Package for Social Sciences (Version 15.0; SPSS, Chicago, IL). The normality of the variables was assessed using histogram charts and Kolmogorov–Smirnov analysis. Considering that, some of variables did not have a normal distribution, we have determined their “log” values and then normalized them. Participants were categorized based on quartiles of LCD. Data on baseline characteristics among participants were expressed according to quartiles of LCD; as the mean ± SD or median (25–75 interquartile range) and percentages for continuous and categorical variables, respectively. We used Chi square and linear regression to test the trend of qualitative and quantitative variables across quartiles of LCD (as median value in each quartile), respectively. The association between LCD scores (overall LCD, animal-based LCD, and plant-based LCD) with diabetes incident were assessed using multivariable logistic regression models and odds ratios (ORs) and 95% confidence interval (CI) were also reported. The potential confounding factors, including age, sex, waist circumference, physical activity, smoking, educational level, daily energy intake, and family history of diabetes were adjusted in multivariable logistic regression models. We have examined the association of confounding variables with the occurrence of diabetes in our population using univariate test. Among these variables, age and waist circumference was significantly associated with type 2 diabetes. P-values < 0.05 were considered to be statistically significant.

## Result

Mean ± SD age and BMI of the participants at baseline (44.4% men) were 40.5 ± 13.0 years and 27.1 ± 4.6 kg/m^2^, respectively. The median (25–75 interquartile range) of the LCD score was 17.0 (12.0–21.0), and after 3 years of follow-up, 123 (2.8%) incident cases of diabetes were ascertained.

Baseline characteristics of the study population across quartiles of LCD score are presented in Table 1. At baseline, individuals with higher LCD score significantly were more likely to be male, low active, younger, had lower frequency of hypertension, lower levels of FPG, HDL-C, SBP, DBP, and TG compared with those in the lowest LCD score (P < 0.05).

**Table 1:** Baseline characteristics of Tehran Lipid and Glucose Study participants based on quartiles of low carbohydrate diet score

Characteristics	Quartiles of low carbohydrate diet score				P for trend
	Q1 (n = 1233)	Q2 (n = 1191)	Q3 (n = 997)	Q4 (n = 935)	
LCD score, Median (minimum–maximum)	9 (3–12)	15 (13–17)	20 (18–21)	24 (22–30)	
Age (years)	41.9 ± 13.6	40.9 ± 13.0	39.6 ± 12.5	39.4 ± 12.4	< 0.001
Male (%)	45.5	50.5	63.2	67.4	< 0.001
High educational level (%)	68.9	68.7	71.1	70.4	0.475
Current smoker (%)	12.6	11.5	10.7	12.3	0.472
Physical activity (MET-h/week)	71.4 (36.4–103.6)	69.9 (34.9–103.2)	61.2 (32.1–95.5)	63.1 (33.7–92.5)	< 0.001
Waist circumference (cm)	92.92	92.76	91.37	91.20	< 0.001
Body mass index (kg/m^2^)	27.1 ± 4.8	27.1 ± 4.5	27.0 ± 4.7	27.4 ± 4.6	0.233
High density lipoprotein-cholesterol (mg/dl)	46.1 ± 11.3	46.8 ± 11.1	48.7 ± 11.6	49.5 ± 11.3	< 0.001
Low density lipoprotein-cholesterol (mg/dl)	112.1 ± 33.0	111.7 ± 32.4	110.1 ± 32.2	110.3 ± 31.8	0.300
Hypertension (%)	15.8	14.0	12.3	13.8	0.025
Triglycerides (mg/dl)	123.0 (86.0–177.0)	119.0 (83.0–171.0)	110.0 (77.0–159.2)	106.0 (77.0–149.0)	< 0.001
Systolic blood pressure (mmHg)	114.2 ± 16.6	113.0 ± 15.5	111.7 ± 15.3	111.8 ± 16.3	< 0.001
Diastolic blood pressure (mmHg)	75.9 ± 11.2	75.6 ± 10.7	74.9 ± 10.7	74.5 ± 11.0	0.006
Fasting blood glucose (mg/dl)	93.1 ± 8.5	92.7 ± 8.5	92.2 ± 8.5	92.1 ± 8.4	0.015
Total cholesterol (mg/dl)	187.0 ± 38.8	186.0 ± 37.2	184.4 ± 37.4	184.5 ± 36.2	0.208

Data are presented as the mean ± SD or as the median (25–75 IQR) for continuous variables and as percentages for categorical variables

The Chi-square and linear regression to test the trend of qualitative and quantitative variables across quartiles of LCD (as median value in each quartile), respectively

Energy-adjusted means for dietary intakes according quartiles of LCD score are expressed in Table 2; dietary intakes of vegetable, nuts, legumes, fish, dairy, and red and processed meat have significantly increased across the quartiles (P < 0.05); however, refined grain, whole grain, fruit intakes, and unsaturated fat/saturated fat have significantly decreased in individuals across quartiles of LCD score (P < 0.05). Also, participants in the highest quartiles of LCD score also had higher intakes of protein, total fat, saturated fat, monounsaturated fat, polyunsaturated fat, and sodium, but had lower intakes of carbohydrate, sucrose, fructose, and total dietary fiber (P < 0.05).

**Table 2:** Dietary intakes of Tehran lipid and glucose study participants based on quartiles of low carbohydrate diet score

Dietary intakes	Quartiles of low carbohydrate diet score				P for trend
	Q1 (n = 1233)	Q2 (n = 1191)	Q3 (n = 997)	Q4 (n = 935)	
Vegetables (g/1000 kcal)	122.6 ± 84.7	131.9 ± 78.4	130.9 ± 77.4	138.7 ± 89.2	< 0.001
Fruits (g/1000 kcal)	237.1 ± 179.3	171.3 ± 113.9	151.3 ± 93.9	129.0 ± 107.7	< 0.001
Legumes and nuts (g/1000 kcal)	19.8 ± 14.4	22.6 ± 15.0	22.9 ± 18.0	24.3 ± 18.8	< 0.001
Refined grains (g/1000 kcal)	211.9 ± 77.5	186.2 ± 56.5	163.3 ± 50.8	137.8 ± 52.1	< 0.001
Whole grains (g/1000 kcal)	33.2 ± 27.0	27.7 ± 23.5	23.0 ± 19.4	19.3 ± 17.7	< 0.001
Dairy (g/1000 kcal)	130.2 ± 68.6	170.4 ± 81.2	183.0 ± 91.9	228.9 ± 113.5	< 0.001
Red and processed meat (g/1000 kcal)	7.2 ± 5.1	8.8 ± 6.2	9.6 ± 7.0	12.6 ± 10.6	< 0.001
Total energy intake (kcal)	2450 ± 739	2373 ± 693	2400 ± 697	2329 ± 700	< 0.001
Protein (% of energy)	11.7 ± 1.6	13.9 ± 3.1	15.2 ± 2.0	17.5 ± 3.4	< 0.001
Carbohydrate (% of energy)	64.7 ± 3.2	58.2 ± 2.7	52.1 ± 9.8	46.8 ± 4.2	< 0.001
Fat (% of energy)	23.6 ± 3.6	27.9 ± 3.3	32.7 ± 5.5	35.7 ± 4.6	< 0.001
Animal Protein (% of energy)	5.2 ± 1.5	6.6 ± 1.9	7.1 ± 2.2	9.2 ± 3.0	< 0.001
Plant Protein (% of energy)	6.5 ± 1.5	7.3 ± 2.0	8.1 ± 2.3	8.3 ± 3.4	< 0.001
Animal Fat (% of energy)	9.6 ± 3.0	12.1 ± 3.2	14.1 ± 4.3	17.0 ± 4.9	< 0.001
Plant Fat(% of energy)	14.0 ± 3.7	15.8 ± 4.5	18.6 ± 6.7	18.7 ± 7.4	< 0.001
Saturated fat (% of energy)	7.6 ± 1.7	9.2 ± 1.9	11.5 ± 5.7	12.1 ± 2.6	< 0.001
Monounsaturated fat (% of energy)	7.8 ± 1.5	9.4 ± 1.8	11.7 ± 5.7	12.1 ± 3.1	< 0.001
Polyunsaturated fat (% of energy)	4.9 ± 1.2	5.7 ± 1.4	7.3 ± 5.8	6.9 ± 2.0	< 0.001
unsaturated fat/Saturated fat	1.74 ± 0.45	1.68 ± 0.44	1.70 ± 0.52	1.62 ± 0.49	< 0.001
Fiber (g/1000 kcal)	21.6 ± 7.2	20.7 ± 8.5	19.1 ± 8.9	17.0 ± 5.7	< 0.001
Sodium (mg/1000 kcal)	1438 ± 431	1521 ± 423	1548 ± 414	1597 ± 497	< 0.001
Sucrose (g/day)	36.5 ± 26.5	31.3 ± 22.8	32.1 ± 22.7	28.2 ± 30.0	< 0.001
Fructose (g/day)	25.0 ± 13.9	20.7 ± 10.7	21.9 ± 8.7	18.0 ± 12.4	< 0.001

Data are presented as the mean ± SD

The association between quartiles of LCD scores and risk of diabetes incident is reported in Table 3. After 3 years of follow-up, a significant positive association was found between overall LCD score and risk of diabetes incident in the highest compared to the lowest quartile, in the age and sex-adjusted model (OR = 1.77, 95% CI:1.05–2.96, P-value = 0.030). In the multivariable-adjusted model, after adjustment for potential confounding variables, including age, sex, smoking status, physical activity, total calorie intake, waist circumference, educational level, and family history of diabetes participants with the highest LCD score had higher odds of incident diabetes, compared to those with the lowest LCD score (OR = 2.16, 95% CI 1.16–4.04, P-value = 0.015). We also have assessed the association of animal- and plant-based LCD score with risk of type 2 diabetes. Based on multivariable adjusted model analysis, the higher score of animal-based LCD was associated with increased risk of type 2 diabetes by 81% (OR = 1.86, 95% CI 1.06–3.11, P-value = 0.029). However, there was no significant association between plant-based LCD score and risk of diabetes incident (OR = 1.47 95% CI 0.85–2.52, P-value = 0.160).

**Table 3:** Odds ratios and 95% CI of type 2 diabetes by low carbohydrate diet scores in the Tehran Lipid and Glucose Study

	Quartiles of low carbohydrate diet score				P-value
	Q1 (n = 1233)	Q2 (n = 1191)	Q3 (n = 997)	Q4 (n = 935)	
Cases n (%)	28 (2.27)	37 (3.10)	23 (2.30)	35 (3.74)	< 0.001
Overall LCD score					
Model 1	1.00 (ref)	1.37 (0.83–2.26)	1.01 (0.58–1.77)	1.67 (1.01–2.76)	0.046
Model 2	1.00 (ref)	1.41 (0.85–2.34)	1.10 (0.61–1.92)	1.77 (1.05–2.96)	0.030
Model 3	1.00 (ref)	1.50 (0.89–2.52)	1.26 (0.67–2.34)	2.16 (1.16–4.04)	0.015
Animal LCD score					
Model 1	1.00 (ref)	1.46 (0.88–2.42)	1.09 (0.63–1.90)	1.56 (0.94–2.60)	0.084
Model 2	1.00 (ref)	1.52 (0.91–2.53)	1.17 (0.67–2.04)	1.68 (1.01–2.82)	0.045
Model 3	1.00 (ref)	1.59 (0.93–2.72)	1.25 (0.70–2.24)	1.81 (1.06–3.11)	0.029
Vegetable LCD score					
Model 1	1.00 (ref)	1.23 (0.73–2.06)	1.28 (0.76–2.16)	1.40 (0.83–2.37)	0.202
Model 2	1.00 (ref)	1.25 (0.74–2.10)	1.33 (0.80–2.25)	1.37 (0.81–2.34)	0.277
Model 3	1.00 (ref)	1.14 (0.66–1.95)	1.30 (0.75–2.22)	1.47 (0.85–2.52)	0.160

Logistic regression models were used to estimate odds ratios (OR) and 95% confidence interval (CI)

Model 1: Crude model

Model 2: Adjusted for age and sex

Model 3: Additionally adjusted for waist circumference, physical activity, educational level, smoking (yes or no), daily energy intake, and family history of diabetes

We have examined the relationship between LCD score and risk of type 2 diabetes in normoglycemia and pre-diabetes groups separately by stratified analysis (Table 4). In pre-diabetes group, after adjustment for confounding factors, higher adherence to LCD score is associated with the increased risk of type 2 diabetes (OR = 2.18 95% CI 1.13–4.20, P-value = 0.019). However, the positive association of higher LCD score with the risk of type 2 diabetes in this group is not statistically significant (OR = 1.85 95% CI 0.66–5.20, P-value = 0.240).

**Table 4:** Odds ratios and 95% CI of type 2 diabetes by low carbohydrate diet scores in normo-glycemia and pre-diabetes groups: a stratified analysis

	Quartiles of low carbohydrate diet score				P-value
	Q1	Q2	Q3	Q4	
Pre-diabetes group	(n = 276)	(n = 235)	(n = 205)	(n = 200)	
Model 1	1.00 (ref)	1.25 (0.67–2.34)	1.16 (0.60 – 2.25)	1.73 (0.94–3.19)	0.077
Model 2	1.00 (ref)	1.24 (0.66–2.34)	1.20 (0.61–2.34)	1.77 (0.95–3.31)	0.071
Model 3	1.00 (ref)	1.40 (0.72–2.79)	1.33 (0.66–2.69)	2.18 (1.13–4.20)	0.019
Normoglycemia group	(n = 956)	(n = 956)	(n = 793)	(n = 735)	
Model 1	1.00 (ref)	1.16 (0.87–5.03)	0.80 (0.27–2.72)	1.87 (0.70–4.93)	0.206
Model 2	1.00 (ref)	1.10 (0.85–5.00)	0.79 (0.24–2.52)	1.64 (0.61–4.40)	0.319
Model 3	1.00 (ref)	1.24 (0.85–5.02)	0.91 (0.27–3.02)	1.85 (0.66–5.20)	0.240

Logistic regression models were used to estimate odds ratios (OR) and 95% confidence interval (CI)

Model 1: Crude model; Model 2: Adjusted for age and sex; Model 3: Additionally adjusted for waist circumference, physical activity, educational level, smoking (yes or no), daily energy intake, and family history of diabetes

## Discussion

The current study provided evidence that greater adherence to LCD was significantly associated with an increased risk of diabetes, especially based on high intakes of protein and fat with an animal source, independent of potential confounding factors in Tehranian adults. However, higher adherence to low carbohydrate diet with higher intakes of plant protein and fat was not related to risk of type 2 diabetes incident.

Evidence on the association of higher adherence to LCD with the risk of type 2 diabetes, lacks sufficient consensus on this topic [10–14]. Similar to our findings, He et al. study has reported that higher adherence to high fat-low carbohydrate diets was associated with a higher risk of type 2 diabetes development [12]. Another study conducted on adult women with GDM also reported that, a low-carbohydrate dietary, particularly with high protein and fat intakes from animal-source foods was positively associated with higher risk of diabetes [13]. Furthermore, findings of the Health Professionals Follow-Up Study with a 20-year follow-up indicated that a greater adherence to LCD high in animal protein and fat was associated with the increased the risk of diabetes in men [10]. Interestingly, in agreement with the findings of our study, the results of two latter studies [10, 13] showed that LCD with high protein and fat intakes from plant-source foods is not significantly associated with the increased risk of diabetes. In contrary to our findings, Halton et al., in a 20-year prospective cohort investigation, reported that a dietary pattern lower in carbohydrate and higher in protein and fat cannot increase the risk of development of type 2 diabetes among women [11]. Moreover, among Japanese population, it was found that a greater adherence to low-carbohydrate dietary pattern was negatively associated with the risk of type 2 diabetes in women [14]. Controversial results of studies on the association between LCD and risk of diabetes may be explained by several reasons. Carbohydrate content of dietary patterns and its food sources in different populations are known as a major source of observed controversies. Iranians consume nearly 60% of energy from carbohydrate, which is higher than the amounts consumed in most of the developed countries [20]. Also, Iranians consume carbohydrate from different food choices such as cereals, fruits, rice, potato, and legumes compared to Western countries [6, 21]. The other reasons to be explained might be differences in gender, age, other individual characteristics, race, duration of follow-up, and adjustment of confounding factors.

Our study, similar to most of previous studies [10, 12, 13], has found that the role of a low-carbohydrate diet in the development of type 2 diabetes depends on the types of protein and fat source. Also our findings revealed that the highest quartile of LCD compared to the lowest quartile of LCD score had higher animal fat (17.0 vs. 9.6 percent of energy) and animal protein (9.2 vs. 5.2 percent of energy). It has been previously reported that higher animal fat intake may result in impaired glucose tolerance and increased risk of diabetes [22, 23]. Also, in some clinical trials, diets rich in animal-derived saturated fatty acids showed higher risk of insulin resistance and glucose intolerance in comparison to plant derived unsaturated fatty acids [24, 25], which in our study, the ratio of unsaturated fats to saturated fats in the highest quartile of LCD score was also less than those in the first quartile of LCD score. Furthermore, the increased total and saturated fat intakes in low carbohydrate- high protein and fat dietary patterns may increase fasting insulin concentrations and could adversely affect glucose metabolism and insulin resistance [26, 27]. In the current study, participants with higher adherence to LCD score tend to have higher intakes of red and processed meat, total protein, and animal protein, which may be associated with increased insulin resistance and diabetes [26, 28]. In comparison to plant protein, higher intake of animal protein in meal can result in higher serum levels of branched-chain amino acids, which have been associated with increased risk of insulin resistance and diabetes in several investigations [29, 30]. Also, dietary score such as LCD rich in red and processed meat and animal protein are positively associated with increased risk of insulin resistance, elevated fasting insulin and glycated haemoglobin, a process that could be mediated through inflammation (hs-CRP), induced by heme–iron and saturated fats in red and processed meats [23, 31–34]. Nitrosamines produced from the interaction between amino compounds with nitrate and nitrite during meat processing or in the preservation of processed meat may be toxic to pancreatic beta cells and increase the risk of diabetes in experimental studies [35, 36]. On the other hand, in our study participants with a high LCD score had lower systolic blood pressure and lower percentage of hypertension than the group with a lower LCD score. It seems that high intake of protein, especially plant protein in the highest quartile of LCD score, has caused this metabolic difference [37, 38].

Our findings indicated that the intake of refined grains has decreased according to the quartiles of LCD score, which could indicate a suitable feature of the LCD diet; however, it should be noted that due to the great reduction in intakes of fruits, whole grain, and dietary fiber across quartiles of LCD score, participants did not have suitable intakes on carbohydrate foods in highest quartile of LCD score. Therefore, these findings support the positive role of LCD score in increasing the risk of type 2 diabetes. A systematic review and meta-analysis reported that individuals with higher fruit intake had an 8% lower risk of incident type 2 diabetes compared to those with lower intake; consumption of 200 g/day of fruits can be effective in prevention of the type 2 diabetes development [39]. In the current study, individuals in the lowest quartile of LCD had higher consumption of fruit (> 230 g/per 1000 kcal/day) compared to those in the highest quartile of LCD (< 130 g/per 1000 kcal/day), therefore, participants in the first quartile of LCD maybe less prone to the occurrence of diabetes. Also, higher consumptions of fruit and whole grain in individuals in the first quartile of LCD led to have higher intake of fiber, polyphenols (such as flavonoids) and antioxidant compounds such as carotenoids, vitamins C, and E. These compounds may decrease risk of type 2 diabetes in subjects of the lowest quartile of LCD by mitigating the oxidative stress, improving endothelial function and insulin sensitivity [39, 40]. In addition, a higher dietary soluble fiber especially from fruit and whole grain may delay the absorption of carbohydrates and consequently inhibit the postprandial glucose load [41]. According to the above mentioned points, progression to type 2 diabetes in individuals, especially those at risk for diabetes can be markedly decreased by lifestyle interventions especially dietary pattern modification as an applicable strategy, this dietary strategy was mostly designed to correct underlying pathophysiological disturbances such as insulin resistance and impaired insulin secretion in a real-world setting [5].

This study had several strengths. The major strengths of the current study were the prospective setting, as well as the relatively large sample size, and the use of the valid and reliable food-frequency and physical activity questionnaires for dietary and physical activity assessments. Despite these strengths, some limitations of the current study should be also reported. The Iranian food composition table was incomplete and the USDA food composition table was mostly used for dietary analyses. Also, it will be difficult to generalize our findings to other societies because of the differences in the dietary behaviors and food intakes of the study participants. Also, despite adjusting of a wide variety of variables in our analysis, residual confounding due to unknown or unmeasured confounders such as inflammatory factors (i.e. hs- CRP, interlukin-6), cannot be excluded.

## Conclusion

In conclusion, to the best of our knowledge, this is the first study conducted in the MENA region that assessed the association of LCD score with the incidence of diabetes. The results provide evidence that a greater adherence to the LCD, with higher intakes of protein and fat from animal foods, was associated with increased risk of type 2 diabetes, however, LCD with consumption of food sources high in plant protein and fat was not related to risk of type 2 diabetes incident. Further clinical studies are required to address the role of LCD diet in the development of type 2 diabetes and its potential mechanisms.

## Data Availability

The datasets analysed in the current study are available from the corresponding author on reasonable request.

## Abbreviations

ADA: American Diabetes Association
BMI: Body mass index
CI: Confidence interval
CVD: Cardiovascular disease
DBP: Diastolic blood pressure
FBS: Fasting blood glucose
FCT: Food composition table
FFQ: Food frequency questionnaire
FPG: Fasting plasma glucose
IR: Insulin resistance
LCD: Low carbohydrate diet
LDL-C: Low density lipoprotein-cholesterol
MAQ: Modifiable Activity Questionnaire
MENA: Middle East and North Africa
MET-h/wk: Metabolic equivalent hours per week
OR: Odds ratios
SBP: Systolic blood pressure
TC: Total cholesterol
TGs: Triglycerides
TLGS: Tehran Lipid and Glucose Study
USDA: United States Department of Agriculture
